# Cost-effectiveness and public health impact of RTS,S/AS01 _E_ malaria vaccine in Malawi, using a Markov static model

**DOI:** 10.12688/wellcomeopenres.16224.1

**Published:** 2020-11-03

**Authors:** Latif Ndeketa, Donnie Mategula, Dianne J. Terlouw, Naor Bar-Zeev, Christophe J. Sauboin, Sophie Biernaux

**Affiliations:** 1 Malawi-Liverpool-Wellcome Trust Clinical Research Programme, College of Medicine, College of Medicine, University of Malawi, Blantyre, Malawi; 2 Liverpool School of Tropical Medicine, Liverpool, L3 5QA, UK; 3 International Vaccine Access Center, Department of International Health, 3. Johns Hopkins Bloomberg School of Public Health, Baltimore, MD 21205, USA; 4 Boehringer Ingelheim Pharma GmbH & Co.KG, Ingelheim am Rhein, D-55216, Germany; 5 Coalition for Epidemic Preparedness Innovations, London, NW1 2BE, UK

**Keywords:** Malaria, Malawi, cost-effectiveness, RTS, S, vaccine, Markov Chain, Modelling

## Abstract

**Background:** The RTS,S/AS01 _E _malaria vaccine is being assessed in Malawi, Ghana and Kenya as part of a large-scale pilot implementation programme. Even if impactful, its incorporation into immunisation programmes will depend on demonstrating cost-effectiveness. We analysed the cost-effectiveness and public health impact of the RTS,S/AS01 _E _malaria vaccine use in Malawi.

**Methods: **We calculated the Incremental Cost Effectiveness Ratio (ICER) per disability-adjusted life year (DALY) averted by vaccination and compared it to Malawi’s mean per capita Gross Domestic Product. We used a previously validated Markov model, which simulated malaria progression in a 2017 Malawian birth cohort for 15 years. We used a 46% vaccine efficacy, 75% vaccine coverage, USD5 estimated cost per vaccine dose, published local treatment costs for clinical malaria and Malawi specific malaria indicators for interventions such as bed net and antimalarial use. We took a healthcare provider, household and societal perspective. Costs were discounted at 3% per year, no discounting was applied to DALYs. For public health impact, we calculated the DALYs, and malaria events averted.

**Results: **The ICER/DALY averted was USD115 and USD109 for the health system perspective and societal perspective respectively, lower than GDP per capita of USD398.6 for Malawi. Sensitivity analyses exploring the impact of variation in vaccine costs, vaccine coverage rate and coverage of four doses showed vaccine implementation would be cost-effective across a wide range of different outcomes. RTS,S/AS01 was predicted to avert a median of 93,940 (range 20,490–126,540) clinical cases and 394 (127–708) deaths for the three-dose schedule, or 116,480 (31,450–160,410) clinical cases and 484 (189–859) deaths for the four-dose schedule, per 100 000 fully vaccinated children.

**Conclusions:** We predict the introduction of the RTS,S/AS01 vaccine in the Malawian expanded programme of immunisation (EPI) likely to be highly cost effective.

## Introduction

Malawi is one of three countries participating in a large-scale pilot implementation programme of the RTS,S AS01 _E_ (GSK) malaria vaccine (henceforth RTS,S) ^1^. Even if impactful, its cost-effectiveness will be a crucial determinant of subsequent introduction ^2^.

RTS,S has been predicted to be highly cost-effective in areas in sub-Saharan Africa with moderate-to-high malaria transmission across different model approaches ^3^. However, health care programmes, vaccination schedules and related cost assumptions vary considerably between LMIC countries. Cognisant of this, national policy makers increasingly seek in-country evidence to inform their decisions. There are no published RTS,S national level cost-effectiveness data for Malawi or for regional countries.

We sought to predict the RTS,S cost-effectiveness and public health impact in Malawi.

## Methods

An intervention is considered cost-effective if the ICER per disability-adjusted life year (DALY) averted is less than three times the GDP per capita and is highly cost effective if the ICER per DALY averted is less than the per capita GDP ^4^.

### Model description

We used a Markov static cohort model developed by GSK for the RTS,S vaccine that has been validated for sub-Saharan Africa; the model is described in depth by Sauboin *et al*. ^5^. The model simulates a birth cohort followed over 15 years under fixed-exposure levels of malaria transmission, taking into account parameters reflecting healthcare provider and societal perspective to calculate the incremental cost effectiveness ratio per DALY averted (ICER) of the RTS,S vaccine ^5^.

**Figure 1.**
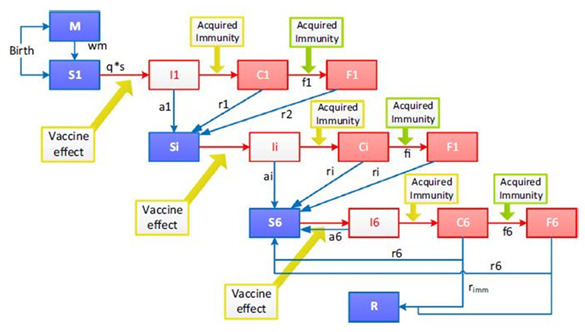
Model structure. The model assumes two processes for acquisition of immunity, one process that protects against clinical malaria of any severity and a faster process that protects against severe malaria. M = maternal protection; S = susceptible; I = infected (parasites emerging from the liver); C = clinical disease episode; F = severe disease episode. There are six levels of immunity with compartments S, I, C and F divided into six levels. R = resistant; wm = waning of maternal immunity; q = probability of infection; s = susceptibility to infection as a function of age; a = probability of asymptomatic infection; r = recovery rate from clinical disease; w = waning rate of acquired immunity; r imm = probability of developing full immunity.

Figure 1 is a diagrammatic representation of the model. The model has compartments susceptible (S), infected (I), clinical disease (C) and severe disease (F) divided into six successive immunity levels following each infection levels.

The model assumes initial protection against malaria from maternal antibodies (M) ^6^. Neonates are considered either protected from (M) or are susceptible to (S1) malaria infection. Initial immunity is presumed to wane exponentially over three months, leaving the child susceptible to infection. An infected (I1) child will have asymptomatic parasitaemia which clears and susceptibility returns (Si), or the child will develop clinical disease (C1). From clinical disease a child may recover (r1) or develop severe disease (F1) where they could either survive returning to a susceptible state or they could die. Immunity is enhanced every level from an asymptomatic state to clinical malaria and to severe disease. The model permits up to six repeated infections to cumulatively increase immunity. Beyond six infections, a fixed proportion of children is assumed to develop a state of resistance (R).

### Model assumptions and inputs

The model uses an estimated 2017 annual birth cohort for Malawi and projected for 15 years ^7^. This birth cohort was the mean of four prior birth cohorts using the United Nations population data ^7^. The model accounts for heterogeneity of individual level exposure and a fixed probability of infection within each transmission category. Malaria transmission intensity in the model was defined categorically as low, medium or high based on *Plasmodium falciparum* parasite prevalence (PfPR) in children aged 2–10 years old of <5%, 5–40% and >40% was respectively, using the Malaria Atlas Project ^8^.

Table 1 shows the input parameters used in the model. The inputs were point estimates extracted from published literature or reasonable assumptions. The vaccine price was based on previously published assumptions since the product has not yet been priced by GSK. The cost of RTS,S vaccine delivery per dose was assumed equal to DTP3 (given as part of pentavalent) in Malawi ^9^. Service delivery make up the bulk (63%) of vaccine delivery costs whilst supply chain and logistics constitute the remainder of vaccine delivery costs ^10^. Vaccine delivery costs mainly comprise of cold chain management, transportation of vaccines to health facilities, waste disposal and additional training for health workers. We sought to calculate cost savings from a healthcare and household perspective. Societal costs are a combination of healthcare and household costs.

**Table 1.** Model inputs.

Parameter	Description and value
Transmission intensity	More than half of the population (54.5%) fell in the moderate intensity category, 42.2% in the high intensity category and only 3.4% were in the low intensity category. Assumed fixed seasonal ^15^
Access to case management	38% ^16^
Bed net coverage	82% own at least one bed net ^16^
Vaccine efficacy	Clinical malaria 46% (95% CI 42% to 50%) Severe malaria 34% (95% CI 15% to 48%)
Vaccine schedule	In Malawi, the first dose of the RTS,S/AS01 vaccine is expected to be administered to children at 5 months with the second and third at one month intervals; the fourth dose is to be given 15–18 months after the third dose.
Vaccine coverage	The coverage of dose 3 of RTS,S was estimated at 75% of DTP3 coverage to account for the challenge in reaching older children and also for the difference in schedule of the RTS,S with traditional vaccines such as the pentavalent vaccine. Dose 4 coverage of RTS,S was 80% of dose 3.
Cost of vaccination	Vaccine delivery costs USD 2.50 ^17^ were estimated from the cost of delivering a single dose of the injectable DTP vaccine.
Clinical malaria estimated costs USD	
Healthcare system	USD 8.02 ^13, 14^. These include drugs, laboratory investigations, staff salaries and facility costs such as laundry, kitchen, sanitation and security.
Household- direct	USD 1.21 ^13, 14^. These include transportation costs, costs of consultations, drugs and diagnostics.
Household- indirect	USD 0.50 ^18^. These are lost income by the carer attributable to the episode of disease
Severe malaria costs USD	
Healthcare system	USD 12.8 ^19^ These include drugs, laboratory investigations, staff salaries and facility costs such as laundry, kitchen, sanitation and security.
Household- direct	USD 14.1 ^14^ These include transportation costs, costs of consultations, drugs and diagnostics.
Household- indirect	USD 4.13 ^14^ These are lost income by the carer attributable to the episode of disease
Sequelae costs USD	
Healthcare system	USD 40.1 ^20^ The neurological sequelae was adapted by the cost of treatment of malaria sequelae for the Tanzanian health system

The Phase III RTS,S/AS01 trial vaccine schedule of 6, 7, 8 and 26 months of age and 18-month follow-up results, following the third dose, were fitted in the model. Vaccine efficacy against clinical and severe malaria in children was 46% (95% CI 42–50%) and 34% (95% CI 15–48%) respectively ^11^. Third and fourth dose RTS,S coverage were assumed to be 75% and 60% of the DTP3 dose 1 coverage respectively. The fourth dose was assumed to restore waning efficacy. Access to artemisinin combination therapy (ACT) or private dispensaries was extracted from the 2014 Malawi Malaria Indicator Survey ^12^.

We used published treatment costs for mild-moderate and severe gastroenteritis, respectively ^13, 14^, since published treatment costs for malaria were outdated or unavailable. These health costs including drugs, laboratory investigations, staff salaries and facility costs. Where these specific costs were unavailable for clinical and severe malaria, we used malaria sequelae costing data from Tanzania ^20^, as cost data from Malawi were not available. Direct and indirect household costs incurred in care seeking were also based on those locally empirically observed in gastroenteritis ^14^. Direct household costs included travel, consultation fees, treatment sought before and after health facility visit and the costs of food and shelter for the carer. Indirect costs comprised income lost while caring for the child ^14^. Bed net use and access to and usage of ACTs and the proportion of those who seek treatment at a private dispensary were derived from the Malawi Malaria Indicator Survey ^12^. Vaccine price per dose has not yet been set by GSK, so we assessed a range of costs of USD1, USD5 and USD10. RTS,S delivery in the Malawi EPI was taken from the administration cost pentavalent vaccine ^2^. The cost of delivery includes all the necessary materials and health worker time required to administer a vaccine in the EPI. The mean Malawi GDP per capita from 2010 to 2015, as reported by the World Bank, was used to compare with the ICER per DALY averted ^21^.

### Sensitivity analysis

Univariate analysis was conducted by running the model through different values of vaccine price and vaccine coverage rate whilst the other input parameters were held constant.

## Results

Based on a 15-year cohort of 711,743 children, the model calculated an ICER of USD 115 and 109 per DALY averted in the health system and the societal perspective respectively compared to no vaccination. Based on a vaccine schedule of four doses, this is less than the Malawi mean GDP per capita of USD 398.6, suggesting that the introduction of RTS,S vaccine to the Malawian EPI programme would be highly cost-effective. The model predicted 721,768 (95% CI: 529,296–894,991) averted clinical malaria cases per year, 14% of current burden. The model demonstrated that 117,260 clinical cases and 700 malaria attributable deaths would be prevented per 100,000 fully vaccinated children per year. The vaccine introduction was also very cost effective at an assumed vaccine price of USD 1 and USD 10 with four doses of the RTS,S vaccine. We predicted cost savings for the society, healthcare system and household as USD 3,025,521, USD 2,433,777 and USD 591,744, respectively. Healthcare costs contributed to over two-thirds of societal costs.

### Modelling findings

Table 2 shows the cumulative cost-effectiveness results for a birth cohort followed up over 15 years, using assumed vaccine prices of USD 1, USD 5, and USD 10. At USD 5, the ICER was 115 USD per DALY averted. At USD 1 vaccine price the ICER was USD 40 per DALY averted and at USD 10 the ICER was USD 209 per DALY averted. We showed that the vaccine would remain very cost-effective even at an inflated vaccine price of USD 10 per dose. However, the societal cost savings remain unchanged with a change in vaccine price.

**Table 2.** Discounted cost-effectiveness results over a 15-year period.

Variable	No vaccination	6–9m schedule plus 4 ^th^ dose			Cost savings ^*^
		USD 1 per dose	USD 5 per dose	USD 10 per dose	
Healthcare system ICER (USD per DALY averted)	---	40	115	209	---
Societal ICER (USD per DALY averted)	---	34	109	202	---
DALYs	1,237,356	1,176,557	1,176,557	1,176,557	96,799
Vaccination costs	---	6,334,532	13,573,997	22,623,329	---
Healthcare system costs	26,396,028	23,962,251	23,962,251	23,962,251	2,433,777
Incremental costs for healthcare system		3,900,755	11,140,220	20,189,552	
Household costs	6,576,176	5,984,432	5,984,432	5,984,432	591,744
Societal costs ^*^	32,972,204	29,946,683	29,946,683	29,946,683	3,025,521
Incremental costs for the society		3,309,011	10,548,476	19,597,808	

DALY = disability-adjusted life year; ICER = Incremental Cost Effectiveness Ratio. Note 1. Societal costs = health care system + household level costs. * Cost savings are the difference in costs between no vaccination scenario and vaccination scenario

Table 3 shows the cumulative public health impact results over 15 years with comparison of different vaccine coverage versus the number of malaria clinical cases averted. The number of DALYs and malaria cases and deaths avoided are largely dependent on the vaccine coverage in the population. At an assumed coverage of 75% of DTP3 coverage, the model predicted 10,826,521 clinical cases averted ( Table 4). This is equal to 721,768 malaria clinical cases per year. The highest number of malaria clinical cases avoided was with a 93% vaccine coverage which is similar to current DTP3 coverage for Malawi. Table 5 shows the comparison in vaccine cost-effectiveness between a three-dose schedule and a four-dose schedule with an assumed vaccine price of USD 5 per dose. It shows that more DALYs are averted with a four-dose schedule than a three-dose schedule, but a four-dose schedule has higher societal costs because of ancillary costs associated with an additional visit.

**Table 3.** Public health impact results cumulative over a period of 15 years.

	Absolute vaccine coverage				
Events averted	93% ^β^	85%	75%	65%	55%
DALYs	120,101	109,706	96,799	83,893	70,986
malaria cases	13,424,866	12,270,057	10,826,521	9,382,985	7,939,449
severe malaria cases	313,359	286,403	252,709	219,014	185,320
malaria hospitalisations	260,329	237,935	209,943	181,950	153,958
malaria deaths	81,824	74,786	65,987	57,189	48,391

β=coverage of DTP3 in Malawi.

**Table 4.** Events averted across different outcome.

	Assessed scenario		
Events averted	Over a 15 year follow up (% reduction compared with no vaccination)	Average per year	per 100,000 vaccines
malaria cases	10,826,521 (14%)	721,768	117,260
severe malaria cases	252,709 (11%)	16,847	2,737
malaria hospitalisations	209,943 (11%)	13,996	2,274
malaria deaths	65,987 (11%)	4,993	714.7

**Table 5.** Comparison of public health impact results over 15 years follow-up between the three- and four-dose schedules.

Events averted	6–9m schedule without a 4 ^th^ dose	6–9m schedule plus a 4 ^th^ dose
DALYs	73,361	96,799
malaria cases	8,504,970	10,826,521
severe malaria cases	200,322	252,709
malaria hospitalisations	166,421	209,943
malaria deaths	52,308	65,987

DALY = disability-adjusted life year.

## Discussion

This analysis has shown that the introduction of the RTS,S vaccine in the Malawi EPI would be a highly cost-effective malaria intervention. Cost-effectiveness of interventions affects decisions to introduce and invest in their sustainable use. Additional economic analyses will further inform budget impact, domestic funding required and long-term financial sustainability of such interventions. With the Markov model, we predicted the incremental cost-effectiveness ratio and public health impact of vaccinating children with four doses of RTS,S as recommended by WHO in the pilot implementation programme.

As the vaccine price is currently unknown, we tested the model at different vaccine prices with other input parameters held constant to determine if the vaccine programme would remain cost-effective. Our results showed that even at an inflated vaccine price of USD 10 per dose, the ICER per DALY calculated was USD 209 suggesting the RTS,S vaccination programme would remain highly cost-effective. We analysed the cost-effectiveness ratio of a three-dose versus a four-dose schedule of the RTS,S vaccine programme. Despite higher vaccine and delivery costs of the four-dose than three-dose schedule, cost-effectiveness is maintained due to greater DALYs averted with the four-dose schedule.

Malawi introduced the Rotavirus vaccine (Rotarix, GSK), in 2012. Similar to RTS,S, Rotarix is a moderately (64%) efficacious vaccine against rotavirus acute gastro-enteritis in Malawi ^22^. A cost-effectiveness analysis in Malawi found it to be highly cost-effective with USD 5.07 ICER per DALY averted with GAVI co-financing and USD 74.73 at vaccine market price ^13^. Rotarix is expectedly more cost-effective than RTS,S as it is delivered in the same schedule and existing vaccine deliver infrastructure as other existing EPI vaccines. The first RTS,S dose will be at 5 months and the last dose at 24 months. This means RTS,S will require a separate immunisation schedule driving the vaccine delivery costs higher. In addition, Rotarix is an oral vaccine with only two doses priced below USD 2.3 per dose whilst RTS,S is an injectable vaccine and has a four-dose schedule with a price assumed to be USD 5 per dose.

The WHO harmonisation exercise on RTS,S cost-effective analysis for sub-Saharan Africa involved four modelling groups: The Institute for Disease Modeling (EMOD-DTK), GSK Vaccines (GSK), Imperial College London (Imperial), and the Swiss Tropical and Public Health Institute (OpenMalaria) ^23^. The EMOD DTK model is a discrete, stochastic, individual-based model for malaria in either local or spatially distributed settings. The model accounts for the combined effect of an extensive set of both vector- and human-directed interventions ^24, 25^. The Imperial College model is a stochastic, individual-based simulation of a single population of humans linked to a stochastic compartmental model for mosquitoes ^26^. The model includes larval stages as well as adult female mosquitoes to capture the feedback of vector control that kills adult mosquitoes in the population dynamics ^27^. Swiss TPH – OpenMalaria is a stochastic, individual-based, single location simulation model of malaria in humans ^28^ linked to a deterministic models of malaria in mosquitoes ^29^. The simulation model includes sub-models of infection of humans ^30^, blood-stage parasite densities ^31^, infectiousness to mosquitoes as a lagged function of asexual parasite density ^32^, incidence of morbidity including severe and hospitalisation and mortality ^33^.

The GSK Markov Model has the advantage of considering the three categories of transmission ( *Pf*PR2<5%, 5≤ *Pf*PR≤40%, *Pf*PR2-10>40%) and capacity to factor in heterogeneity in exposure among individuals for each transmission level. This model does not report confidence bounds, which is expected from a cohort model nor can it account for possible herd protection. The GSK cohort-based model was the optimal option as the other models use individual level data which are unavailable in Malawi. Our findings were within the confidence bounds of 116,480 (31,450–160,410) clinical malaria cases averted per 100,000 vaccinated children as predicted by the other three models. The GSK model is unable to capture the effect of herd immunity as compared to the three other models which do. Should herd immunity occur, cost-effectiveness would be greater than our predictions. The modest efficacy of RTS,S and its short duration of protection may limit its potential for reducing the parasite circulation capacity. Our method provides point estimates but not 95% confidence bounds, the latter which require microsimulation on individual data which were unavailable to us. The fourth dose of RTS,S was assumed to restore waning immunity but recent data has shown the efficacy to be lower after dose four.

Models are input dependent. Cost data in Africa are sparse, may be out of date or insufficiently robust. Regional data or neighbouring country data may be used when available. A malaria cost of illness study in Tanzania, Kenya and Ghana estimated clinical malaria and severe malaria costs for household and the healthcare system ^34^. Where Malawian data were unavailable, we used Tanzanian data rather than data from Ghana or Kenya. This is because the Tanzanian and Malawian health financing systems are similar, both provide government funded free health care through primary and referral level systems, and both lack a national insurance system or any substantial private health sector ^35^. Additionally, direct household cost for clinical malaria was more similar for Malawi (USD 0.5) and Tanzania (USD 0.4) than it was for Kenya (USD 0.7) and Ghana (USD 4.4) Malawi and Tanzania are geographically contiguous and share similar malaria epidemiology.

In the absence of published malaria treatment cost data from the societal perspective, we used rotavirus empirical cost data. Our data on bed net usage, an important model parameter, was taken from the 2014 Malaria Indicator Survey ^12^ which preceded the national wide bed net campaign that that distributed over 2.3 million bed nets from November 2014 to February 2015 ^16^. The RTS,S vaccine is a complementary malaria intervention whose impact on the reduction malaria morbidity is also dependent on the coverage of other interventions such as bed net usage.

Population coverage is crucial to the success of any vaccine programme ^36^. RTS,S will be given to older children, aged 5 months, not as part of the standard EPI schedule. Additional, the fourth dose of the RTS,S will be administered to children when they are about 2 years of age. This booster dose will be outside the normal immunisation schedule whilst the first 3 doses will be before the measles vaccine which is given at 9 months. In our study we assumed the RTS,S dose 3 coverage at 75% of DTP3 and the fourth dose to be even lower at 80% of RTS,S of the third dose ^37, 38^. The coverage rate for the measles vaccine has been above 80% since 2010 ^37, 38^ even though the vaccine is given to older children. Almost all Malawians have been affected by malaria, which may translate to high vaccine acceptance despite the non-standard schedule.

## Conclusion

Introduction of the RTS,S/AS01 vaccine would be a highly cost-effective malaria intervention in Malawi. This holds regardless of potential changes to key variables for the vaccine programme. Following full recommendation of vaccine use by WHO, individual level cost-effective analyses will provide more accurate data that can assist other sub-Saharan African countries.

## Data availability

All data underlying the results are available as part of the article and no additional source data are required.
