# Supplementary material for: Cost-effectiveness and public health impact of RTS,S/AS01 E malaria vaccine in Malawi, using a Markov static model
Source: Wellcome Open Res. 2021 Aug 12;5:260. Originally published 2020 Nov 3. [Version 2] doi: 10.12688/wellcomeopenres.16224.2 (PMC8491149; doi:10.12688/wellcomeopenres.16224.2)
Supplement: Peer review history

# Peer review: Reviewer response for version 2

**DOI:** 10.21956/wellcomeopenres.18918.r45415

**Published:** 2021-10-04

**Authors:** Liriye Kurtovic

**Affiliations:** 1 Department of Immunology and Pathology, Monash University, Burnet Institute, Melbourne, Victoria, Australia

I approve the revised version of the manuscript.

Is the work clearly and accurately presented and does it cite the current literature?

Yes

If applicable, is the statistical analysis and its interpretation appropriate?

I cannot comment. A qualified statistician is required.

Are all the source data underlying the results available to ensure full reproducibility?

Yes

Is the study design appropriate and is the work technically sound?

Yes

Are the conclusions drawn adequately supported by the results?

Yes

Are sufficient details of methods and analysis provided to allow replication by others?

Yes

Reviewer Expertise:

Malaria; Immunology; Vaccines

I confirm that I have read this submission and believe that I have an appropriate level of expertise to confirm that it is of an acceptable scientific standard.

# Peer review: Reviewer response for version 1

**DOI:** 10.21956/wellcomeopenres.17820.r43392

**Published:** 2021-04-26

**Authors:** Suraj Chawla

**Affiliations:** 1 Department of Community Medicine, Shaheed Hasan Khan Mewati Government Medical College, Nalhar, Haryana, 122107, India

The authors analysed the cost-effectiveness and public health impact of the RTS,S/AS01 _E _malaria vaccine use in Malawi and predicted that introduction of the RTS,S/AS01 vaccine in the Malawian expanded programme of immunisation (EPI) to be highly cost-effective. The cost-effectiveness of vaccines affects decisions to introduce and invest in their sustainable use. Hence, the study findings are highly relevant in this regard.

 I would like to give some suggestions for the authors to consider:

1. The introduction section is very short; they could have included the magnitude of malaria in Malawi in terms of morbidity and mortality data. It would have been better if they had provided information regarding vaccine efficacy, acceptance, and other relevant phase III trial data in the introduction.
2. The authors have used a range of vaccine price per dose, similarly they could have used a range of vaccine efficacy available from existing literature to calculate Incremental Cost-Effective Ratio (ICER), Disability-adjusted Life Year (DALYs) averted, and cost savings. Then it would have been easier for decision-makers to know the cost-effectiveness for a wide range of vaccine efficacy.

Is the work clearly and accurately presented and does it cite the current literature?

Yes

If applicable, is the statistical analysis and its interpretation appropriate?

Yes

Are all the source data underlying the results available to ensure full reproducibility?

Yes

Is the study design appropriate and is the work technically sound?

Yes

Are the conclusions drawn adequately supported by the results?

Yes

Are sufficient details of methods and analysis provided to allow replication by others?

Yes

Reviewer Expertise:

Vaccinology; Epidemiology; Health system research

I confirm that I have read this submission and believe that I have an appropriate level of expertise to confirm that it is of an acceptable scientific standard.

# Response

**Published:** 2021-07-30

**Authors:** Latif Ndeketa

**Affiliations:** Malawi Liverpool Wellcome Trust Clinical Research Programme, Malawi

Thank you so much for taking your time to review this paper. Your comments were relevant and thoughtful and we appreciate the improvements they have made to the paper.

 “The introduction section is very short; they could have included the magnitude of malaria in Malawi in terms of morbidity and mortality data. It would have been better if they had provided information regarding vaccine efficacy, acceptance, and other relevant phase III trial data in the introduction.”

**Response:** This has been added

 “The authors have used a range of vaccine price per dose, similarly they could have used a range of vaccine efficacy available from existing literature to calculate Incremental Cost-Effective Ratio (ICER), Disability-adjusted Life Year (DALYs) averted, and cost savings. Then it would have been easier for decision-makers to know the cost-effectiveness for a wide range of vaccine efficacy.

**Response:** Thank you for this comment. We used is a cohort model and intrinsically the efficacy declines over time so the cost effectiveness and public health impact calculated account for that

# Peer review: Reviewer response for version 1

**DOI:** 10.21956/wellcomeopenres.17820.r42239

**Published:** 2021-02-19

**Authors:** Liriye Kurtovic

**Affiliations:** 1 Department of Immunology and Pathology, Monash University, Burnet Institute, Melbourne, Victoria, Australia

Ndeketa et al., analyzed the cost-effectiveness and public health benefit of implementing the RTS,S/AS01 malaria vaccine in Malawian children. They authors found that the ICER/DALY averted was lower than the GDP per capita, supporting that RTS,S implementation is highly cost effective and beneficial in reducing malaria clinical cases and deaths. Given that RTS,S is currently being evaluated in a pilot implementation program, these findings are highly relevant and timely, and may contribute to further decision-making regarding the wider implementation of RTS,S.

 I have concerns regarding the assumption that VE over 15 years is 46% against clinical malaria. i) In the phase 3 trial, 46% VE was calculated in children who received three vaccine doses over an 18-month follow-up. Why was the overall VE used and not the VE from the Malawi study site (Lilongwe), which was 42%? ii) The Methods state that the 4th dose was assumed to restore VE. However, in the phase 3 trial, VE in children who received the 4th dose was only 36% after a 4-year follow-up. Therefore, the booster dose did not restore VE (which continued to wane since the 18-month follow-up). Further, other trials have shown RTS,S vaccine efficacy to rapidly wane within years after immunization (Olotu et al., N Eng J Med 2016 ^1^). iii) There is no evidence that RTS,S vaccine efficacy remains moderate at 46% for 15 years. These are incredibly important limitations to the model presented. I would suggest the assumed VE needs to be revised, or this limitation must be further emphasised in the discussion section (along with relevant published data on the true longevity of vaccine efficacy over time).

 I have several minor comments for the authors to consider:

1. The introduction section is very brief and would benefit from additional background discussion on the RTS,S malaria vaccine. In particular, important information from the phase 3 trial as the model is based on a 4-dose vaccine regimen (which was tested in the phase 3 trial) and vaccine efficacy against clinical/severe malaria were also based on data from the phase 3 trial (in children).
2. Figure 1 appears quite blurred, could a higher-resolution image be uploaded?
3. The Methods “Sensitivity analysis” subsection says that univariate analysis was performed – are these data shown? If not, this should be specified.

Please note that I do not have expertise in modelling or cost-effective analysis and cannot confirm the analysis and interpretation are appropriate. However, the model has been previously validated in a peer-reviewed manuscript.

Is the work clearly and accurately presented and does it cite the current literature?

Yes

If applicable, is the statistical analysis and its interpretation appropriate?

I cannot comment. A qualified statistician is required.

Are all the source data underlying the results available to ensure full reproducibility?

Yes

Is the study design appropriate and is the work technically sound?

Yes

Are the conclusions drawn adequately supported by the results?

Yes

Are sufficient details of methods and analysis provided to allow replication by others?

Yes

Reviewer Expertise:

Malaria; Immunology; Vaccines

I confirm that I have read this submission and believe that I have an appropriate level of expertise to confirm that it is of an acceptable scientific standard, however I have significant reservations, as outlined above.

## References

[1] : Seven-Year Efficacy of RTS,S/AS01 Malaria Vaccine among Young African Children.*N Engl J Med*.2016;374(26) : 10.1056/NEJMoa15152572519-2910.1056/NEJMoa151525727355532PMC4962898

# Response

**Published:** 2021-07-30

**Authors:** Latif Ndeketa

**Affiliations:** Malawi Liverpool Wellcome Trust Clinical Research Programme, Malawi

Dear Liriye Kurtovic,

 Thank you so much for taking your time to review this paper. Your comments were relevant and thoughtful and we appreciate the improvements they have made to the paper.

 “Why was the overall VE used and not the VE from the Malawi study site (Lilongwe), which was 42%?” **Response: ** 

 As the phase III trial was a multicenter trial (11 centers), using Malawi specific efficacy would be invalid as it would not be representative of the sample size which was necessary to detect a difference in protection between the vaccinated group and the control group.

 “The Methods state that the 4th dose was assumed to restore VE. However, in the phase 3 trial, VE in children who received the 4th dose was only 36% after a 4-year follow-up”

 Response:

 “There is no evidence that RTS,S vaccine efficacy remains moderate at 46% for 15 years.”

 Response:

 “The introduction section is very brief and would benefit from additional background discussion on the RTS,S malaria vaccine. In particular, important information from the phase 3 trial as the model is based on a 4-dose vaccine regimen (which was tested in the phase 3 trial) and vaccine efficacy against clinical/severe malaria were also based on data from the phase 3 trial (in children).”

 Response: This has been amended

 “Figure 1 appears quite blurred, could a higher-resolution image be uploaded?”

 Response: This has been corrected

 “The Methods “Sensitivity analysis” subsection says that univariate analysis was performed – are these data shown? If not, this should be specified.”

 Text has been added to indicate that the results of the sensitivity analyses are in tables 3 and 4

 “There is no evidence that RTS,S vaccine efficacy remains moderate at 46% for 15 years.”

 Response: Thank you for the comment, the model follows a stochastic process following a birth cohort for 15 years. This does not indicate the vaccine efficacy of the vaccine remains protective for 15 years. We have rephrased this sentence to clear any misunderstanding

 “The Methods state that the 4th dose was assumed to restore VE. However, in the phase 3 trial, VE in children who received the 4th dose was only 36% after a 4-year follow-up.:

 Response: Thank you for this comment. We have rephrased the sentenced to reflect what the 4 ^th^ dose does i.e boosting waning immunity
